# New methods for assessing secondary performance attributes of sunscreens suitable for professional outdoor work

**DOI:** 10.1186/s12995-021-00314-2

**Published:** 2021-07-05

**Authors:** Marc Rocholl, Patricia Weinert, Stephan Bielfeldt, Sabrina Laing, Klaus Peter Wilhelm, Claas Ulrich, Swen Malte John

**Affiliations:** 1 Institute for Health Research and Education, Department of Dermatology, Environmental Medicine and Health Theory, University of Osnabrück, Am Finkenhügel 7a, 49076 Osnabrück, Germany; 2 Institute for Interdisciplinary Dermatological Prevention and Rehabilitation (iDerm) at the University of Osnabrück, Am Finkenhügel 7a, 49076 Osnabrück, Germany; 3 proDERM Institute of Applied Dermatological Research GmbH, Kiebitzweg 2, 22869 Schenefeld, Hamburg Germany; 4 Department of Dermatology and Allergy, Skin Cancer Center, Charité-Universitätsmedizin, Charitéplatz 1, 10117 Berlin, Germany

**Keywords:** Sunscreening agents, Sun protection factor, Ultraviolet rays, Outdoor work, Photoprotection, Occupational health, Environmental health, Skin neoplasms

## Abstract

**Background:**

Outdoor workers (OW) are highly exposed to solar ultraviolet radiation (UVR) and thus at increased risk for developing skin cancer. An essential part of an overall strategy to reduce workplace UVR-exposure to OW’s skin is the usage of sunscreens. However, compliance with regular sunscreen usage seems to be low, as products are usually designed for recreational sun exposure and thus do not meet the requirements of physically active OW. To date, no standardized test procedures assess the suitability of sunscreens for professional use. The aim of this pilot study was to develop standardized methods of testing secondary performance attributes (PA) to represent real-life working conditions of outdoor work.

**Methods:**

Ten sunscreen products, carefully selected after a detailed market survey of all relevant producers available on the German market, were evaluated regarding their suitability for professional outdoor work on 24 healthy volunteers in a newly designed test procedure. In addition to three standardized efficacy characteristics, i.e., sun protection factor, water-resistance, and UVA protection, we evaluated each PA involving parameters typically associated with outdoor workplaces.

**Results:**

We developed standardized methods for objectifying the suitability of sunscreen products for professional outdoor work. The test procedures used are well feasible and appropriate for testing the PA because they represent practical working conditions in detail – although the degree of discriminability of single test methods varied. The claimed sun protection factor (SPF) of the products was confirmed; bio-stability of the SPF after physical activity was achieved in most cases. While most products hardly irritate the eyes and are quickly absorbed, the evaluation of the subjective skin feeling and non-slip grip is inconsistent.

**Conclusions:**

In this pilot study, for the first time secondary PA are defined and examined. Although further objectification of the PA assessment as well as the establishment of minimum standards should be sought, the new methods could already complement the so far mandatory labels and in this way provide a significant impetus for the current scientific and political focus on the improvement of occupational health in highly UVR-exposed OW.

**Supplementary Information:**

The online version contains supplementary material available at 10.1186/s12995-021-00314-2.

## Background

Solar ultraviolet radiation (UVR) is a significant risk factor for developing non-melanoma skin cancer (NMSC) [1] – more precisely referred to as keratinocyte carcinoma (KC) [2]) – and has legitimately been classified as carcinogenic to humans (Group 1) by the *International Agency for Research on Cancer* (*IARC*) [3]. In the European Union, about 14.5 million outdoor workers (OW) are highly exposed to solar UVR, as they spend at least 75% of their working time outside [4–6], and thus are at increased risk for developing NMSC [7]. This applies to construction workers, roofers, fishermen, police officers, farmers, gardeners, ski instructors, lifeguards, and many more alike [8]. The association between cumulative UVR exposure, which is characteristic for outdoor work, and basal cell carcinoma (BCC) [9–11], cutaneous squamous cell carcinoma (cSCC)) [12, 13] as well as intra-epidermal precursor lesions such as actinic keratoses (AK, in situ cSCC) [14, 15] is scientifically proven. In Europe, a number of countries have therefore recognized cSCC as an occupational disease, although this does not consistently apply to AK, BCC, or malignant melanoma (MM) [16].

Besides the recommended implementation of technical and organizational sun-protective measures (e.g., adjusting working hours, using solar sails to provide shade) as well as wearing protective clothing and brimmed hats, the application of sunscreens with a high sun protection factor (SPF) is an important measure for covering unprotected areas of the skin (e.g., forehead, ears, neck, hands). This can be considered as an essential part of an overall strategy to reduce workplace UVR exposure to OW’s skin [17–19]. Studies suggest that the regular use of sunscreens can help reduce skin damage from natural UVR [20–22]. However, compliance with regular sunscreen usage still seems to be lacking amongst OW [23–25]. In addition to behavioral barriers (e.g. forgetfulness [23, 26]), the acceptance of the products is often low due to inappropriate secondary performance attributes of sunscreens that do not meet the requirements of an outdoor workplace. Several studies [23, 25–31] report that OW commonly perceive sunscreen as greasy, sticky, and overall uncomfortable. Ideally, the products used for professional outdoor work should have the following attributes: fast-absorbing, non-greasy, non-sticky, easy to apply, not eye irritating, sweat resistant, not dust adhering, not soiling clothes, not impairing manual work, avoiding slipperiness and loss of safe grip [23, 25–32].

As most sunscreens are made for leisure time, as for example beach holidays, doing sports, taking care of kids, or sensitive skin, an outdoor workplace however brings different requirements (such as climatic conditions and sustained physical activities, e.g. at a construction site or in agriculture). The suitability assessment of products, therefore, needs to be based on different criteria. While the efficacy of sunscreens and the related approval for the European market is usually expressed by means of three performance characteristics (SPF, water-resistance, and UVA/UVB protection), all of which are objectively evaluated either in vivo or in vitro according to international test methods [33–35], additional secondary performance attributes of sunscreen products for professional outdoor workplaces (see Table 1) are neither mandatory nor yet testable in a standardized manner.

**Table 1:** Secondary performance attributes representing real-life working conditions of outdoor workplaces according to [32]

	Performance attribute (PA)
**PA 1**	Bio-stability on the skin
**PA 2**	Eye irritation (burning)
**PA 3**	Absorption time
**PA 4**	Grip and subjective skin feeling
**PA 5**	Compatibility with textiles
**PA 6**	Dust and dirt absorption
PA 7^*^	Whitening effect

PA = Performance attribute

^*^ During the course of the study, it was noticed that after the application of some test products white product residues remained on the skin (so-called “*whitening effect*”). Therefore, this PA was subsequently included in the study, even though the criterion is not directly derivable from the literature

Therefore, the aim of the present study was (1) to develop and pilot-test feasibility of standardized methods of testing secondary performance attributes to represent real-life working conditions of outdoor work as well as (2) to determine the extent to which 10 products, carefully selected by a detailed market survey including all producers available on the German market with a relevant market share, comply with the specific requirements for outdoor work.

## Methods

### Study design

This pilot study was conducted at and in cooperation with the *proDERM Institute for Applied Dermatological Research*, an independent institute for clinical studies, Schenefeld/Hamburg, Germany, from February to April 2019. For each performance attribute (see Table 1), a study design was developed by modifying already established or developing new test procedures. Each test was conducted according to an approved study protocol following the principles of *Good Clinical Practice* (GCP) as a guide of reference. All examinations were performed in accordance with the principle requirements of the Declaration of Helsinki [36], taken into account to protect the rights, safety, and well-being of subjects participating in the study. All study participants provided written informed consent to participate and were informed that withdrawal of their consent at any time would not lead to any disadvantages. The study was approved by the Institutional Review Board of the *proDERM Institute for Applied Dermatological Research*.

### Study subjects

Twenty-four healthy volunteers (79% female, *n* = 19) between 18 and 70 years of age (mean age (M): 55.0 years; standard deviation (SD): ±12.0 years) with skin phototype I, II, and III according to Fitzpatrick [37] participated in our study. Inclusion and exclusion criteria are listed in detail in Table AF 1 (see Additional File 1).

### Tested products

In order to define the test products, we conducted a market analysis on sunscreen products commercially available on the German market. Producers with a relevant market share in Germany were explicitly invited to identify products within their companies’ portfolios that would best comply with the above-defined PA (see Table 1). Mandatory requirements for products were also an SPF > 50, proven UVA protection, and being water-resistant. Products that either are in the higher price segment (over 30 Euro), are explicitly advertised for women (e.g., tinted products), are only available in high-quality perfumeries, or are not sold on the German market were excluded. In total, 9 out of 27 producers with a relevant market share in Germany responded to our questionnaire survey and reported on 38 of their products. The evaluation of the questionnaires revealed that producers rarely test any of the relevant secondary PA systematically. It can therefore be assumed that these are not considered in the development process of sunscreens. Finally, based on the producers’ claims regarding their products, according to the best fit model, ten out of 38 products were selected to be tested regarding their suitability for outdoor work. Of these ten, eight were cosmetic products (from the low-price segment to higher-value products) and two were medicinal products. Five products were labeled as water-resistant or very water-resistant. Details on galenic characteristics and UV filters contained in the products are provided in Additional File 2 (see Table AF 2). The rational selection of products was screened and approved by the interdisciplinary research advisory group of the project.

### Sunscreen efficacy and water-resistance

The efficacy of the products was examined by means of three mandatory performance attributes: in vivo determination of the SPF according to ISO 24444 [33], in vitro determination of sunscreen UVA photo-protection according to ISO 24443 [34, 38], and determination of water-resistance in accordance with the Colipa Guidelines for Evaluating Sun Product Water-resistance [35].

### PA 1: bio-stability on the skin

The *bio-stability on the skin* when physically active, i.e., resistance to sweat, was determined by examining the SPF of the products after induced sweating on a gym stepper. According to a randomization scheme, 2 mg/cm^2^ (± 0.05 mg/cm^2^) test product was applied to test areas of at least 30 cm^2^ and a maximum of 60 cm^2^ on the back of the subjects by a technician. Fifteen minutes after product application (absorption time), all test persons underwent a physical activity on a gym stepper for some 40 min until sweat droplets were seen on the back. After a subsequent rest period of at least 1 hour, the exercise was repeated for additional 40 min. The procedures took place in an air-conditioned room at a temperature of approximately 25 °C (± 2 °C). Four hours (± 30 min) after product application on the back, all test areas were irradiated with a sun simulator (300 W Multiport Solar Simulator, Solar Light Company, Philadelphia, PA, USA) approximating the ISO 24444 [33]. The visual rating of irradiated skin by a trained technician was performed 16 to 24 h after irradiation. The minimal erythema dose (MED) was defined as the lowest UVR dose that produces the first perceptible unambiguous erythema with defined borders appearing over most of the field of UVR exposure. Skin spot evaluation was observer-blinded. Hence, the observer was not the same person as the one who applied the products and conducted the irradiation, so the observer was not aware of the randomization of sites and UVR-doses.

### PA 2: eye irritation when sweating (burning)

The experimental set-up for assessing the *eye tolerability* of the test products after sweating was carried out together with the PA 1 test. For this purpose, an amount of 2 mg/cm^2^ (± 0.05 mg/cm^2^) was applied on one half of each subject’s face (randomly assigned to the left and right side of the face) with a Finnpipette (Thermo Scientific™, Bremen, Germany) by a technician. Subjects themselves evenly distributed the test products on the assigned half-face. The absorption time prior to the 40 min of physical activity was 15 min. Immediately after the first round (see PA 1), the subjects assessed feeling of burning in the eyes on a 5-point scale: 0 = “*no burning*”, 0.5 = “*very slight burning*”, 1 = “*slight burning*”, 2 = “*moderate burning*”, 3 = “*strong burning*”. Afterwards, the subjects washed their face with an unscented standard soap provided. The procedure was repeated in the second run. Since only three products per subject were tested, one-half face remained untreated.

### PA 3: absorption time

The *absorption time* was investigated by application of products on the back of the hands and assessment whether the test product had been absorbed at three different time points. Before applying the test products, subjects were asked to wash their hands according to a standard washing procedure with a mild liquid soap. After a drying time of 10 minutes, only one test product at a time was applied by a technician on the hands with a smart dosing applicator. Assuming that both hands (palm of the hands) equal approx. 300 cm^2^, an amount of 600 mg was used to finally end up with a thickness layer of 2 mg/cm^2^. The subjects rubbed the test products between the palms of the hands until evenly distributed for approximately 20 s. Absorption of the test product was assessed after 1, 2, and 3 minutes (yes: “*test product is absorbed*”; no: “*test product is not absorbed*”) and, if applicable, 10 minutes if not yet absorbed after 3 minutes. Absorption time data was evaluated by reporting the counts and percentage of “*yes*” at each assessment time.

### PA 4: grip and subjective skin feeling

The experimental set-up for assessing the *non*-*slip grip and skin feeling* of the products was carried out together with the PA 3 test. Three minutes after standardized product application and evenly spreading the test materials, subjects held a smooth wooden bar (resembles a tool, e.g., hammer shaft) and a smooth metal bar (resembles metal scaffold tube), one in each hand, to evaluate the slip resistance and evaluate the tactile feel on the skin. Assessments were made on a 5-point Likert scale: 1 = “*very good skin feeling / very good grip*”, 2 = “*good skin feeling / good grip*”, 3 = “*moderate skin feeling / moderate grip*”, 4 = “*unpleasant skin feeling / slippery*”, 5 = “*very unpleasant skin feeling / very slippery*”.

### PA 5: compatibility with textiles

According to a randomization scheme, approx. 2 mg/cm^2^ (± 0.05 mg/cm^2^) of the test products (equals approx. 2 μL/cm^2^) was applied on marked areas (approx. 24 cm^2^) of the back by a technician with a Finnpipette. For retrieval of the test areas and to prevent cross-contamination, plaster strips were affixed and additionally marked with a black pen. After 15 min of absorption time, the subjects wore a white T-shirt provided to them for about 4 hours (± 30 min). The study participants were allowed to leave the research institute and engage in everyday activities during this time, with the exception of intense physical activity involving heavy sweating. After 4 hours (± 30 min), the T-shirt was removed and pictures were taken with a camera directly installed under a Wood Lamp to visualize the rub off of the test products, which was visible as a dark spot on the inner sides of the shirts. Three trained graders evaluated rub off on the pictures using a 5-point Likert scale: 1 = “*no rub off*”, 2 = “*very slight rub off*”, 3 = “*slight rub off*”, 4 = “*moderate rub off*”, 5 = “*high rub off*”.

### PA6: dust and dirt absorption

The evaluation of *dust and dirt absorption* was carried out in accordance with the existing sand resistance test developed by Caswell et al. [39]. Five pre-defined test areas (approx. 50 cm^2^) on the back were marked and test products (approx. 2 mg/cm^2^; ± 0.05 mg/cm^2^) were applied according to a randomization scheme. After an absorption time of 15 min, 30 to 40 ml of oven-dried (approx. 24 h at 90 °C) fine sand were poured from a height of approximately 10 cm above onto the test areas by a technician until they were completely covered while the subject was in a prone position. Five minutes after sand application, the subject stood up and the test areas were slightly brushed with a 2.5 cm paintbrush to remove loosely attached sand particles. Photos of the subject’s back were taken with a camera at a standardized distance. These photos were assessed by three trained graders using a 5-point Likert scale: 1 = “*no dust absorption*”, 2 = “*very slight dust absorption*”, 3 = “*slight dust absorption*”, 4 = “*moderate dust absorption*”, 5 = “*high dust absorption*”.

### PA 7: whitening effect

Approximately 2 mg/cm^2^ (± 0.05 mg/cm^2^) of each test product was applied on the back of one study participant (approx. 24 cm^2^) and a picture was taken after 15 min of absorption time. Two trained graders independently ranked the white product residues that remained on the skin on a scale from 1 to 5 with 1 being “*no whitening*” and 5 being “*highest whitening*”.

### Statistical analysis

Descriptive data analysis was performed using SAS for Windows. Mean, standard deviation, median, minimum (Min), and maximum (Max) were calculated.

## Results

### Sunscreen efficacy and sun product water-resistance

For all tested products, the claimed SPF of 50(+) could be confirmed. Mean SPF values ranged from SPF 54.3 to 83.2. Results of in-vitro determination of UVA photoprotection showed sufficient UVA protection for six products. Four products did not achieve UVA photoprotection in accordance with ISO 24443 [34] and the Commission Recommendation of the European Union of 22 September 2006 [38]. Altogether, the UVA protection factors (UVA-PF) among products varied widely, between UVA-PF 11 and 35 (see Table 2). Except for one product (P3), (very) water-resistance was confirmed. Results are shown in Table 2.

**Table 2:** Results of UVA photoprotection determination and determination of UVB water-resistance

	P 1	P 2	P 3	P 4	P 5	P 6	P 7^**†**^	P 8^**†**^	P 9	P 10
**In-vitro UVA photoprotection (ISO 24443** **[**34**])**										
**Ratio**_(≤ 3)_	3.3	4.1	1.7	5.2	2.2	1.9	4.5	2.5	1.9.	2.4
**UVA-PF**_(SPF in vivo/Ratio)_	23.8	14.5	38.2	11.5	37.5	28.6	17.3	25.7	35.2	34.7
**Critical Wavelength**^**#**^_≥370 nm_	374.5	375	381.2	372.7	377.5	379.5	376.6	379.5	381.5	378.8
**Sufficient UVA protection**	**x**	**x**	**✓**	**x**	**✓**	**✓**	**x**	**✓**	**✓**	**✓**
**Colipa Guidelines for Evaluating Sun Product Water-resistance**^‡^ **[**35**]**										
	*very water-resistance*					*water-resistance*				
**In-vivo SPF**_(according to_ [33]_)_	78.5	59.6	64.9	59.8	82.6	54.3	77.7	64.2	66.8	83.2
**SPF**_after water immersion procedure_	45.0	41.0	26.2	37.1	57.4	50.2	58.3	42.1	49	45.3
**(very) water-resistance**	**✓**	**✓**	**x**	**✓**	**✓**	**✓**	**✓**	**✓**	**✓**	**✓**

^†^ = Medicinal product

# = The critical wavelength is defined as the wavelength for which the area below the absorbance curve comprises 90% of the total absorbance curve. According to ISO 24443 [34] and the Commission Recommendation of the European Union [38], the critical wavelength has to be equal or higher than 370 nm in order to claim “broad spectrum protection”

‡ = The evaluation of water-resistance was carried out with *n* = 6 participants. For all products, producers had claimed full UVA photoprotection and UVB water resistance

### PA 1: bio-stability on the skin

**Fig. 1:**
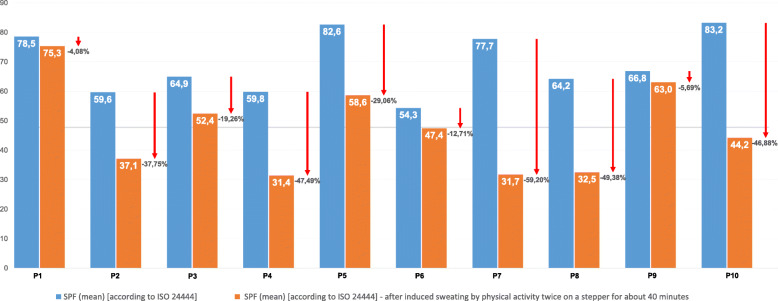
Stability of the SPF after physical activity. Legend: The blue bars show the initial SPF according to ISO 24444, the orange ones show the bio-stability of the SPF after twice-induced sweating by physical activity on a gym stepper for 40 min. The arrows represent the decrease in percentage on the SPF. For all products, producers had claimed full bio-stability after sweating

The in-vivo bio-stability of the products after prolonged absorption time and repeated sweating is achieved but to a varying degree. The mean SPF values decreased by between 4.08 and 59.2% (see Fig. 1). While mean SPF values of six test products are below the respective labeled SPF of 50(+) (ranging between SPF 31.4 and 47.4), four test products are still at the same level as the labeled value, with the highest SPF mean value of 75.3.

### PA 2: eye irritation when sweating (burning)

The results of the subjective *eye irritation* evaluation are presented in Table 3.

**Table 3:** Evaluation of subjective eye irritation (burning sensation) after physical activity

	Eye burning sensation											
	***no burning***		***very slight***		***slight***		***moderate***		***strong***		***Total***	
	n	%	n	%	n	%	n	%	n	%	n	sum score
**P1**	9	75	1	8	1	8	1	8	0	0	12	3.5
**P2**	11	92	1	8	0	0	0	0	0	0	12	0.5
**P3**	10	77	0	0	1	8	2	15	0	0	13	5
**P4**	9	75	2	17	1	8	0	0	0	0	12	2
**P5**	8	67	1	8	1	8	0	0	2	17	12	7.5
**P6**	10	77	2	15	1	8	0	0	0	0	13	2
**P7**^†^	9	75	1	8	2	17	0	0	0	0	12	2.5
**P8**^†^	9	75	1	8	1	8	0	0	1	8	12	4.5
**P9**	11	85	1	8	0	0	1	8	0	0	13	2.5
**P10**	8	62	0	0	3	23	2	15	0	0	13	7

Score: 0 = “no burning”, 0.5 = “very slight burning”, 1 = “slight burning”, 2 = “moderate burning”, 3 = “strong burning”

† = Medicinal product

### PA 3: absorption time

In the majority of cases, products were absorbed between 1 and 2 minutes. An absorption time of up to 10 minutes was reported by at least one participant for six out of the ten tested products. Absorption time data are shown in detail in Table 4 and Fig. AF 1 (see Additional File 1).

**Table 4:** Absorption time data by counts and percentages of “*yes*” at assessment time

	Absorption Time							
	*1 min*		*2 min*		*3 min*		*10 min*	
	n	%	n	%	n	%	n	%
P1*	16	70	1	4	6	25	0	0
**P2**	16	67	6	25	2	8	0	0
**P3**	10	42	7	29	7	29	0	0
P4*	16	70	2	9	5	22	0	0
P5*	12	52	6	26	1	4	4	17
**P6**	12	50	9	38	2	8	1	4
**P7**^†^	9	38	7	29	5	21	2	8
**P8**^†^	20	83	2	8	0	0	2	8
**P9**	11	46	6	25	5	21	2	8
**P10**	11	46	7	29	5	21	1	4

* = data of 1 subject is missing

† = Medicinal product

### PA4: grip and subjective skin feeling

In terms of skin feeling, almost no differences were detected between the test products or between the wooden and metal bar. Evaluation of grip on the wooden bar revealed that one product (P8) was rated with almost 96% favorable statements (includes the answers for “*very good grip*”, “*good grip*”, and “*moderate grip*”). In contrast, products P3, P5, and P9 were rated as unfavorable (includes the answers for “*slippery*” and “*very slippery*”) by a large proportion of the subjects (P3: 41.7%; P5: 50.0%; P9: 50.0%). Regarding the assessment of grip on metal, products P5 and P9 were again rated as unfavorable (P5: 58.3%; P9: 41.7%). A large majority of participants rated product 2 favorably (87.5%). Results are shown in detail in Figs. AF 2 – AF 5 (see Additional File 1).

### PA 5: compatibility with textiles

Results show no clear differences between the products. As mean values vary between 3.43 (SD = 0.56) and 4.19 (SD = 0.45), in all cases slight to moderate rub off was detected.

### PA 6: dust and dirt absorption

Only one product (P8) was assessed by the trained graders as not dust absorbing or only slightly dust absorbing with a mean value of 1.67 (SD = 0.68). There was no substantial difference observed in all other products as graders rated them between “*slight dust absorption*” and “*moderate dust absorption*”: mean values range from 2.63 (SD = 0.79) to 3.97. (SD = 0.61).

### PA 7: whitening effect

No whitening effect at all was found for three products (P3, P6 & P9), while two products were rated with the highest whitening effect on the skin (P5 & P10). Results are summarized in Table 5. Both graders rated identical.

**Table 5:** Evaluation of whitening effect

	Evaluation of whitening effect
**P 1**	3
**P 2**	2
**P 3**	1
**P 4**	4
**P 5**	5
**P 6**	1
**P 7**^†^	2
**P 8**^†^	4
**P 9**	1
**P 10**	5

1 = “no whitening”, 5 = “highest whitening”

† = Medicinal product

## Discussion

The present study aimed to develop and pilot-test feasibility of new laboratory test procedures to evaluate secondary performance attributes of sunscreens suitable for professional outdoor work, which so far have not been taken into consideration when testing sunscreen efficacy. In addition, it should be determined to what extent ten selected sunscreen products of producers with a relevant market share comply with these requirements and producers’ claims.

The usage of sunscreen is an important part of an overall strategy in the prevention of occupationally induced skin cancer, although technical and organizational sun-protective measures, as well as clothing and headgear, should be given priority [17–19]. However, studies have shown that, in practice, technical and organizational measures are often highly influenced by the general conditions in the workplace and, therefore are rarely implemented in most cases [23, 26, 40]. The use of personal protective equipment (e.g., long-sleeved clothes, headgear) is also often inadequate [23, 24]. Sunscreens should – as a secondary measure – therefore just add to other protective measures and should especially be applied to areas of the body that cannot be protected with clothing (e.g., forehead, ears, neck, and hands). It needs to be emphasized, however, that AK, cSCC and BCC in outdoor workers predominantly occur in exactly these body areas [10–12, 41]. Thus, these body areas are of pivotal importance for adequate photo-protection and skin cancer prevention at workplaces. Furthermore, this highlights the need of sunscreens that meet the specific requirements for outdoor work.

To ensure effective protection against UVR, current guidelines recommend a sunscreen with a high SPF and sufficient UVA photo-protection [17–19]. In our study, we confirmed the claimed SPF of the products, in some cases with SPF clearly higher than 50 (see Fig. 1). Nevertheless, it has to be mentioned that these results are not entirely transferable to the application in practice since we used a sunscreen thickness layer of 2 mg/cm^2^. Under real-life conditions, however, the applied amount is often significantly lower, which leads to a significant decrease in protective performance [27, 42–44]. On the one hand, this underlines the necessity of recommending a high SPF; on the other hand, it emphasizes the need to train OW in correct usage, ideally with targeted teaching strategies [18, 43]. Furthermore, we demonstrated the bio-stability of the SPF after physical activity – although to a varying degree. Overall, the bio-stability of the very water-resistant products (P1 - P5) was on average more stable than that of the water-resistant products (P6 - P10), even though for one product, the label “very water-resistant” could not be confirmed. At this point, however, it should be taken into account that the evaluation of water-resistance was carried out with 6 participants and not with 10, as required by the Colipa Guideline [35]. This might limit the validity of our results. These results are similar to the findings of the investigation by Bodekaer et al. [45], who examined the effect of physical activity, heat and bathing – in other words, a simulated day at the beach – on the stability of the SPF in an organic and an inorganic sunscreen. For the practical application, these results indicate that the re-application of sunscreen products every 2 hours in sufficient quantities – with the aim of maintaining the SPF – is of particular importance. According to a recent randomized-controlled trial by Matta et al. [46], which received considerable media attention, the repeated application of sunscreens on 75% of the body area with 2 mg/cm^2^ led to systemic resorption and increased overall plasma concentration of active ingredients (e.g., between 3.3 ng/mL and 7.1 ng/mL for avobenzone). Although the transferability of these findings to the application of sunscreen in practice is a matter for discussion [47–49], the authors encourage use due to the evident protective effects of sunscreens [46]. Nevertheless, this once again underlines the importance of first-line sun-protective measures (e.g., brimmed hats, wearing long-sleeved clothes) to reduce blood transmission by vastly limiting application areas.

The aim of this pilot study was to develop new methods for assessing secondary PA that best mimic working conditions of outdoor workplaces (‘*workplace simulation*’). Therefore, both a wooden and a metal bar were used to evaluate subjective skin feeling and grip (see PA 4). These bars served to simulate specific parts of working materials or tools – e.g., a wooden hammer shaft vs. a metal scaffold tube. This PA is – among others – essential in the construction industry since secure grip is crucial from an occupational safety point of view. Closely related to this PA – and therefore tested together – is the absorption time of a sunscreen product (PA 3), which can influence safety aspects, but is also a key factor for user acceptance (e.g., through galenic properties). While the aim of our pilot study was to best represent real-life working conditions, it is questionable to what extent the laboratory tests are equal to an assessment by OW in a workplace context. Bauer et al. [27], for example, carried out a randomized-controlled, cross-over trial in the workplace and evaluated the overall acceptance of the daily use of two sunscreens. The examination of our laboratory results by OW in practice, therefore, seem feasible and might be an important step for validation and quality assurance.

Regarding limitations of our piloting study, the lack of minimum requirements for the PA test procedures should be mentioned. Therefore, a final overall appraisal of the tested products – except for an ordinal ranking, which is a straightforward approach but would be of limited informative value - is only possible to a limited extent. The definition of minimum standards, − i.e., which scores should be achieved in the given tests – is, however, a complex process of consensus building that must be carried out involving different normative institutions. Future studies – once this ongoing process is completed – can consider the minimum requirements and in this fashion provide a ranking of the most suitable sunscreens for professional outdoor work. As this has not yet been included in our pilot study, a weighting of the PA should in addition be considered to prevent selected PA having a too strong influence on an overall estimation. In future studies, the weighting of our suggested PA could also be industry-specific, since their relevance may be varying according to the work environment. Furthermore, prospectively, an interactive ‘*online tool*’ that allows to weight PA in order to match them to a specific industry could be useful (e.g., very high water resistance for bath attendants, dust and dirt absorption as well as non-slip grip for the construction industry or agriculture). This would facilitate the selection of sunscreens, for both, employers, and employees, and enhance acceptance by the social partners. In addition, it could be useful to establish a certified label (e.g., ‘*suitable for the professional sector*’). Social accident insurances, ministries of labour or bodies responsible for safety and health at work, could establish such an industry standard.

Another limitation of the present study is the experimental setting, which may only be transferable to the daily practice of the broad spectrum of practical outdoor workplace settings to a limited extent. In particular, the use of 2 mg/cm^2^ sunscreen does not match the applied amount of sunscreen in practice [42, 43]. The measured SPF, as well as the bio-stability, is therefore probably lower under practical conditions. On the other hand, the amount of sunscreen used leads to rather conservative estimates, especially regarding the secondary PAs (e.g., absorption time and subjective skin feeling). Under real-life working conditions, paradoxically, it might thus be expected that acceptance will be somewhat higher than in the laboratory tests.

Although the experimental setting of our pilot study implies a high degree of standardization, studies that involve human subjects are always prone to a certain variability. This becomes apparent, for example, in the experimental conditions of PA 5 (‘compatibility with textiles’). Since the subjects were allowed to leave the research institute during the investigation, it cannot be completely ensured that they always followed the instructions of the study personnel. One further aspect that limits the transferability of our results is that the assessments of the PA are based on external observers or the statements of the study participants rather than on objective (instrumental) measurements. Even though the assessments were carried out by at least two trained external graders, an objectification through instrumental measurement procedures would be of advantage and could contribute to the reproducibility of our results. Especially for PA 4 (evaluation of grip) or PA 7 (whitening effect), instrumental measurements (e.g., usage of Chromameter assessments or color measurement of image analysis for whitening effect; friction measurements for the evaluation of grip) might be possible. The development and validation of additional objective assessment methods is currently under further consideration; however, subjective assessments cannot be dispensed for PA 2 (eye irritation) and PA 4 (subjective skin feeling).

A strength of the present pilot study is that the development of the PA – except for PA 7 (whitening effect) – was derived from the literature. The assessment of the whitening effect was the only one that was subsequently added during the investigations. In our pilot study, the whitening effect was assessed by two trained observers based on a photo of a subject’s back. Since the extent to which white residue is detected on the skin may be influenced by the skin color of the subject to whose back the products were applied, the results must be interpreted with caution. Nevertheless, it should be noted that the results of the two graders in our study are highly consistent. In future studies, the extent of the whitening effect should be determined with more test subjects in order to be able to exclude confounding influences, e.g., skin color. In addition, a reference product could also be used to facilitate the assessment. Moreover, by using a Chromameter device or color image analysis, an objective measurement method could be of advantage.

The selection of participants for this pilot study focused on eligible subjects who were also able to perform sensory grading with a satisfying quality. In our pilot study, we have a sex imbalance, as most of the participants were women. Future studies should therefore include a larger proportion of men in the test panel to increase representability in terms of the intended target population, although – regarding the very heterogeneous group of OW – representability strongly depends on the respective (industrial) sector.

Overall, the test methods are feasible and seem to be suitable for testing secondary PAs, although the degree of discriminability of the single test methods varied. For example, there were barely any differences in the raw data for PA 2, PA 5, and PA 6. Yet while only carefully selected products according to their producers’ claims were tested, it is all the more remarkable that half of the products did not meet their producers’ claims.

The strength of this study lies in its unique approach and design as until to date no standard test method has been developed to assess other PA than the three mandatory efficacy statements. To our knowledge, this study has identified for the first time a specific test-battery for objectifying the suitability of appropriate sunscreen formulations and thus provided a significant impetus for the current scientific and political focus on improvement of occupational health in highly UVR-exposed OW [50–52]. Our study aims to open up a discussion on new testing methods focusing on outdoor workers’ expectations and the intended scope of application that complement the so far mandatory labels and assist in making informed, evidence-based decisions when choosing an appropriate sunscreen product. Furthermore, for manufacturers there now rises a significant opportunity to fulfill this demand by manufacturing quality, efficacious and safe sunscreen formulations specifically targeted for outdoor workers.

## Conclusions

The tests for important secondary PA, which were defined and examined for the first time by this pilot study and are yet to be further standardized, could, in addition to the three existing mandatory efficacy statements, establish the suitability of a sunscreen for OW in a targeted manner. Further objectification of the PA assessment as well as the establishment of minimum standards are the subject of an ongoing process. With an increasing awareness of the population to protect their skin against UVR and consumers’ diverse preferences of a sunscreen, the demand for improved sunscreen formulations will presumably increase invariably. Hence, the new testing methods are an important prerequisite to further develop a new generation of sunscreens optimized for the use of OW for primary prevention of sunburn, actinic damage, and skin cancer.

## Supplementary Information

**Additional file 1.** Table AF 1: Inclusion and exclusion criteria for study participation. Fig. AF 1: Evaluation of absorption time. Absorption time was assessed 1, 2, and 3 min after test product application and, if applicable, 10 min after application, if not yet absorbed after 3 min. Data show percentages of “yes” ratings at assessment time. Single subjects stated an absorption time of 10 min regarding products P5 – P 10 while none of the subjects stated an absorption time longer than 3 min for codes P1, P2, P3 and P4. The shortest time to be absorbed was found for code P8. Fig. AF 2: Evaluation of subjective evaluation of skin feeling on wood. Subjective evaluation of skin feeling on a wooden bar after an absorption time of 3 min. Participants rated subjective skin feeling on a 5-point Likert scale. Favorable: Includes the answers for “very good skin feeling”, “good skin feeling” and “moderate skin feeling”. Unfavorable: Includes the answers for “unpleasant skin feeling” and “very unpleasant skin feeling”. Fig. AF 3: Evaluation of subjective evaluation of skin feeling on metal. Subjective evaluation of skin feeling on a metal bar after an absorption time of 3 min. Participants rated subjective skin feeling on a 5-point Likert scale. Favorable: Includes the answers for “very good skin feeling”, “good skin feeling” and “moderate skin feeling”. Unfavorable: Includes the answers for “unpleasant skin feeling” and “very unpleasant skin feeling. Figure AF 4: Evaluation of non-slip grip on wood. Subjective evaluation of non-slip grip on a wooden bar after an absorption time of 3 minutes. Participants rated subjective grip on a 5-point Likert scale. The best assessment for grip 3 minutes after product application was achieved by product P8 (about 96% favorable answers). Lowest frequencies of favorable answers were obtained for products P5, P9 and P3 (between 50% and 58% favorable answers). Favorable: Includes the answers for “very good grip”, “good grip” and “moderate grip”. Unfavorable: Includes the answers for “slippery” and “very slippery“. Figure AF 5: Evaluation of non-slip grip on metal. Subjective evaluation of non-slip grip on a metal bar after an absorption time of 3 minutes. Participants rated subjective grip on a 5-point Likert scale. The best assessment for grip 3 minutes after product application was achieved by product P2 (about 88% favorable answers). Lowest frequencies of favorable answers were obtained for products P5 and P9 (between 42% and 58% favorable answers). Favorable: Includes the answers for “very good grip”, “good grip” and “moderate grip”. Unfavorable: Includes the answers for “slippery” and “very slippery”.

**Additional file 2.** Table AF 2: Overview of tested products.

## Data Availability

The datasets used and analyzed during the current study are available from the corresponding author on reasonable request.

## Abbreviations

AK: actinic keratoses
BCC: basal cell carcinoma
Colipa: *European Cosmetic and Perfumery Association*
cSCC: cutaneous squamous cell carcinoma
GCP: Good Clinical Practice
IARC: *International Agency for Research on Cancer*
KC: keratinocyte carcinoma
M: mean
MED: Minimal Erythema Dose
MM: malignant melanoma
nm: nanometer
NMSC: non-melanoma skin cancer
OW: outdoor workers
PA: performance attribute
SD: standard deviation
SPF: sun protection factor
UVA: ultraviolet radiation in the spectrum 320–400 nm
UVA-PF: UVA protection factor
UVB: ultraviolet radiation in the spectrum 190–320 nm
UVR: ultraviolet radiation

## References

[1] Armstrong BK, Kricker A (2001). The epidemiology of UV induced skin cancer. J Photochem Photobiol B.

[2] Karimkhani C, Boyers LN, Dellavalle RP, Weinstock MA (2015). It's time for "keratinocyte carcinoma" to replace the term "nonmelanoma skin cancer". J Am Acad Dermatol.

[3] World Health Organization (WHO). Radiation: Volume 100 D - a Review of Human Carcinogens. Lyon: International Agency for Research on Cancer; 2012.

[4] European Agency for Safety and Health at Work (2009). Outlook 1 -New and emerging risks in occupational safety and health; European risk observatory.

[5] Wittlich M, John SM, Tiplica GS, Sălăvăstru CM, Butacu AI, Modenese A, Paolucci V, D'Hauw G, Gobba F, Sartorelli P, Macan J, Kovačić J, Grandahl K, Moldovan H (2020). Personal solar ultraviolet radiation dosimetry in an occupational setting across Europe. J Eur Acad Dermatol Venereol.

[6] Moldovan HR, Wittlich M, John SM, Brans R, Tiplica GS, Salavastru C, Voidazan ST, Duca RC, Fugulyan E, Horvath G, Alexa A, Butacu AI (2020). Exposure to solar UV radiation in outdoor construction workers using personal dosimetry. Environ Res.

[7] Trakatelli M, Barkitzi K, Apap C, Majewski S, de Vries E (2016). Skin cancer risk in outdoor workers: a European multicenter case-control study. J Eur Acad Dermatol Venereol.

[8] Diepgen TL, Fartasch M, Drexler H, Schmitt J (2012). Occupational skin cancer induced by ultraviolet radiation and its prevention. Br J Dermatol.

[9] Bauer A, Diepgen TL, Schmitt J (2011). Is occupational solar ultraviolet irradiation a relevant risk factor for basal cell carcinoma? A systematic review and meta-analysis of the epidemiological literature. Br J Dermatol.

[10] Bauer A, Haufe E, Heinrich L, Seidler A, Schulze HJ, Elsner P (2020). Basal cell carcinoma risk and solar UV exposure in occupationally relevant anatomic sites: do histological subtype, tumor localization and Fitzpatrick phototype play a role? A population-based case-control study. J Occup Med Toxicol.

[11] Schmitt J, Haufe E, Trautmann F, Schulze H-J, Elsner P, Drexler H, Bauer A, Letzel S, John SM, Fartasch M, Brüning T, Seidler A, Dugas-Breit S, Gina M, Weistenhöfer W, Bachmann K, Bruhn I, Lang BM, Bonness S, Allam JP, Grobe W, Stange T, Westerhausen S, Knuschke P, Wittlich M, Diepgen TL, FB 181 Study Group (2018). Occupational UV-exposure is a major risk factor for basal cell carcinoma: results of the population-based case-control study FB-181. J Occup Environ Med.

[12] Schmitt J, Haufe E, Trautmann F, Schulze H-J, Elsner P, Drexler H, Bauer A, Letzel S, John SM, Fartasch M, Brüning T, Seidler A, Dugas-Breit S, Gina M, Weistenhöfer W, Bachmann K, Bruhn I, Lang BM, Bonness S, Allam JP, Grobe W, Stange T, Westerhausen S, Knuschke P, Wittlich M, Diepgen TL, for the FB-181 Study Group (2018). Is ultraviolet exposure acquired at work the most important risk factor for cutaneous squamous cell carcinoma? Results of the population-based case-control study FB-181. Br J Dermatol.

[13] Schmitt J, Seidler A, Diepgen TL, Bauer A (2011). Occupational ultraviolet light exposure increases the risk for the development of cutaneous squamous cell carcinoma: a systematic review and meta-analysis. Br J Dermatol.

[14] Rosen T, Lebwohl MG (2013). Prevalence and awareness of actinic keratosis: barriers and opportunities. J Am Acad Dermatol.

[15] Berman B, Cockerell CJ (2013). Pathobiology of actinic keratosis: ultraviolet-dependent keratinocyte proliferation. J Am Acad Dermatol.

[16] Ulrich C, Salavastru C, Agner T, Bauer A, Brans R, Crepy MN, Ettler K, Gobba F, Goncalo M, Imko-Walczuk B, Lear J, Macan J, Modenese A, Paoli J, Sartorelli P, Stageland K, Weinert P, Wroblewski N, Wulf HC, John SM (2016). The European status quo in legal recognition and patient-care services of occupational skin cancer. J Eur Acad Dermatol Venereol.

[17] International Commission on Non-Ionizing Radiation Protection (2010). ICNIRP statement -protection of workers against ultraviolet radiation. Health Phys.

[18] Bauer A, Beissert S, Knuschke P (2015). Prävention von durch berufliche solare UV-exposition bedingtem epithelialem Hautkrebs. Hautarzt..

[19] Krebsgesellschaft D, Krebshilfe D, Leitlinienprogramm Onkologie (AWMF) (2020). S3-Leitlinie Prävention von Hautkrebs, Langversion 2.01 (Konsultationsfassung).

[20] Green AC, Williams GM, Logan V, Strutton GM (2011). Reduced melanoma after regular sunscreen use: randomized trial follow-up. J Clin Oncol.

[21] Hughes MCB, Williams GM, Baker P, Green AC (2013). Sunscreen and prevention of skin aging: a randomized trial. Ann Intern Med.

[22] Ulrich C, Jürgensen JS, Degen A, Hackethal M, Ulrich M, Patel MJ, Eberle J, Terhorst D, Sterry W, Stockfleth E (2009). Prevention of non-melanoma skin cancer in organ transplant patients by regular use of a sunscreen: a 24 months, prospective, case-control study. Br J Dermatol.

[23] Reinau D, Weiss M, Meier CR, Diepgen TL, Surber C (2013). Outdoor workers' sun-related knowledge, attitudes and protective behaviours: a systematic review of cross-sectional and interventional studies. Br J Dermatol.

[24] Ziehfreund S, Schuster B, Zink A (2019). Primary prevention of keratinocyte carcinoma among outdoor workers, the general population and medical professionals: a systematic review updated for 2019. J Eur Acad Dermatol Venereol.

[25] Kearney GD, Xu X, Balanay JAG, Becker AJ (2014). Sun safety among farmers and farmworkers: a review. J Agromedicine.

[26] Rocholl M, Ludewig M, John SM, Bitzer EM, Wilke A (2020). Outdoor workers' perceptions of skin cancer risk and attitudes to sun-protective measures: a qualitative study. J Occup Health.

[27] Bauer A, Hault K, Püschel A, Rönsch H, Knuschke P, Beissert S (2014). Acceptance and usability of different sunscreen formulations among outdoor workers: a randomized, single-blind, cross-over study. Acta Derm Venereol.

[28] Lee C, Duffy SA, Louzon SA, Waltje AH, Ronis DL, Redman RW, Kao T-S (2014). The impact of sun solutions educational interventions on select health belief model constructs. Workplace Health Saf.

[29] Woolley T, Buettner PG, Lowe J (2002). Sun-related behaviors of outdoor working men with a history of non-melanoma skin cancer. J Occup Environ Med.

[30] Malak AT, Yildirim P, Yildiz Z, Bektas M (2011). Effects of training about skin cancer on farmers' knowledge level and attitudes. Asian Pac J Cancer Prev.

[31] Weber M, Uller A, Schulmeister K, Brusl H, Hann H, Kindl P (2007). Outdoor workers' acceptance of personal protective measures against solar ultraviolet radiation. Photochem Photobiol.

[32] Heerfordt IM, John SM, Wulf HC, Ulrich C. Sunscreen use and attitude towards use among outdoor workers - a systematic review. Manuscript in preparation.

[33] International Organization for Standardization. ISO 24444:2010–11: Cosmetics - Sun protection test methods - In vivo determination of the sun protection factor (SPF).

[34] International Organization for Standardization. ISO 24443:2012–06: Determination of sunscreen UVA photoprotection in vitro.

[35] Cosmetics Europe (2005). Colipa guidelines for evaluating sun product water resistance.

[36] World Medical Association (2013). World medical association declaration of Helsinki: ethical principles for medical research involving human subjects. JAMA..

[37] Fitzpatrick TB (1988). The validity and practicality of sun-reactive skin types I through VI. Arch Dermatol.

[38] Commission of the European Communities. Comission Recommendation of 22 September 2006 on the efficacy of sunscreen products and the claims made relating thereto (2006/647/EC). Official Journal of the European Union. 2006:L 265/39.

[39] Caswell M, Wood C, Martinez A (2012). Sand resistance of sunscreens. J Cosmet Sci.

[40] Knuschke P, Ott G, Janßen M, Mersiowsky K-P, Püschel A, Rönsch H (2014). Die neue BK 5103 “Hautkrebs” - Notwendigkeit und Möglichkeiten der Primärprävention. Ergebnisse aus dem BAuA-Forschungsprojekt F 2036 [the new occupational disease BK 5103 “skin cancer” - needs of primary prevention and ways of realization. Results of the BAuA research project F 2036]. Derm Beruf Umwelt.

[41] Weber A, Tizek L, Biedermann T, Zink A (2020). High-risk body sites for actinic keratosis in outdoor and indoor workers: a retrospective review. J Am Acad Dermatol.

[42] Faurschou A, Wulf HC (2007). The relation between sun protection factor and amount of suncreen applied in vivo. Br J Dermatol.

[43] Petersen B, Wulf HC (2014). Application of sunscreen--theory and reality. Photodermatol Photoimmunol Photomed.

[44] Bimczok R, Gers-Barlag H, Mundt C, Klette E, Bielfeldt S, Rudolph T, Pflücker F, Heinrich U, Tronnier H, Johncock W, Klebon B, Westenfelder H, Flöβer-Müller H, Jenni K, Kockott D, Lademann J, Herzog B, Rohr M (2007). Influence of applied quantity of sunscreen products on the sun protection factor--a multicenter study organized by the DGK task force sun protection. Skin Pharmacol Physiol.

[45] Bodekaer M, Faurschou A, Philipsen PA, Wulf HC (2008). Sun protection factor persistence during a day with physical activity and bathing. Photodermatol Photoimmunol Photomed.

[46] Matta MK, Florian J, Zusterzeel R, Pilli NR, Patel V, Volpe DA, Yang Y, Oh L, Bashaw E, Zineh I, Sanabria C, Kemp S, Godfrey A, Adah S, Coelho S, Wang J, Furlong LA, Ganley C, Michele T, Strauss DG (2020). Effect of sunscreen application on plasma concentration of sunscreen active ingredients: a randomized clinical trial. JAMA..

[47] Sander M, Sander M, Burbidge T, Beecker J (2020). The efficacy and safety of sunscreen use for the prevention of skin cancer. CMAJ..

[48] Fox JD, Benesh G, Abrouk M, Kirsner RS (2020). Controversies in sunscreens: a practical approach. Am J Med.

[49] Adamson AS, Shinkai K (2020). Systemic absorption of sunscreen: balancing benefits with unknown harms. JAMA..

[50] John SM, Garbe C, French LE, Takala J, Yared W, Cardone A, et al. Improved protection of outdoor workers from solar ultraviolet radiation: position statement. J Eur Acad Dermatol Venereol. 2020. 10.1111/jdv.17011. 10.1111/jdv.17011 33222341

[51] European Commission. Europe's beating Cancer plan: a new EU approach to prevention, treatment and care. 2021. https://ec.europa.eu/health/sites/health/files/non_communicable_diseases/docs/eu_cancer-plan_en.pdf. Accessed 8 Mar 2021.

[52] Garbe C, Peris K, Soura E, Forsea AM, Hauschild A, Arenbergerova M, Bylaite M, Marmol V, Bataille V, Samimi M, Gandini S, Saiag P, Eigentler TK, Lallas A, Zalaudek I, Lebbe C, Grob JJ, Hoeller C, Robert C, Dréno B, Arenberger P, Kandolf-Sekulovic L, Kaufmann R, Malvehy J, Puig S, Leiter U, Ribero S, Papadavid E, Quaglino P, Bagot M, John SM, Richard MA, Trakatelli M, Salavastru C, Borradori L, Marinovic B, Enk A, Pincelli C, Ioannides D, Paul C, Stratigos AJ (2020). The evolving field of Dermato-oncology and the role of dermatologists: position paper of the EADO, EADV and task forces, EDF, IDS, EBDV-UEMS and EORTC cutaneous lymphoma task force. J Eur Acad Dermatol Venereol.

