# Supplementary material for: New methods for assessing secondary performance attributes of sunscreens suitable for professional outdoor work
Source: J Occup Med Toxicol. 2021 Jul 5;16:25. doi: 10.1186/s12995-021-00314-2 (PMC8256554; doi:10.1186/s12995-021-00314-2)
Supplement: Supplementary file 1 — Additional file 1. Table AF 1: Inclusion and exclusion criteria for study participation. Fig. AF 1: Evaluation of absorption time. Absorption time was assessed 1, 2, and 3 min after test product application and, if applicable, 10 min after application, if not yet absorbed after 3 min. Data show percentages of “yes” ratings at assessment time. Single subjects stated an absorption time of 10 min regarding products P5 – P 10 while none of the subjects stated an absorption time longer than 3 min for codes P1, P2, P3 and P4. The shortest time to be absorbed was found for code P8. Fig. AF 2: Evaluation of subjective evaluation of skin feeling on wood. Subjective evaluation of skin feeling on a wooden bar after an absorption time of 3 min. Participants rated subjective skin feeling on a 5-point Likert scale. Favorable: Includes the answers for “very good skin feeling”, “good skin feeling” and “moderate skin feeling”. Unfavorable: Includes the answers for “unpleasant skin feeling” and “very unpleasant skin feeling”. Fig. AF 3: Evaluation of subjective evaluation of skin feeling on metal. Subjective evaluation of skin feeling on a metal bar after an absorption time of 3 min. Participants rated subjective skin feeling on a 5-point Likert scale. Favorable: Includes the answers for “very good skin feeling”, “good skin feeling” and “moderate skin feeling”. Unfavorable: Includes the answers for “unpleasant skin feeling” and “very unpleasant skin feeling. Figure AF 4: Evaluation of non-slip grip on wood. Subjective evaluation of non-slip grip on a wooden bar after an absorption time of 3 minutes. Participants rated subjective grip on a 5-point Likert scale. The best assessment for grip 3 minutes after product application was achieved by product P8 (about 96% favorable answers). Lowest frequencies of favorable answers were obtained for products P5, P9 and P3 (between 50% and 58% favorable answers). Favorable: Includes the answers for “very good grip”, “good grip” and “moderate grip [file 12995_2021_314_MOESM1_ESM.docx]

Additional File 1

**Table AF 1:** Inclusion and exclusion criteria for study participation.

| **Inclusion criteria** | **Exclusion criteria** |
| --- | --- |
| female and/or male | female subjects: Pregnancy or lactation |
| between 18 and 70 years of age | conditions which exclude a participation or might influence the test reaction/evaluation such as concomitant medications having the potential to interfere with the study (e.g. anti-inflammatories and photosensitizing drugs) |
| written Informed Consent to participate in the study | AIDS, HIV positive or infectious hepatitis if known to the subjects |
| healthy skin in the test areas | drug addicts, alcoholics |
| willingness to actively participate in the study and to come to the scheduled visits | participation or being in the waiting period after participation in similar cosmetic and/or pharmaceutical studies |
|  | documented allergies to sunscreen products |
|  | active skin disease at the test area |
|  | any topical medication at the test areas |
|  | irregularly tanned skin on the test areas |
|  | regular use of tanning beds |
|  | moles, tattoos, scars, irritated skin, hairs, etc. at the test areas that could influence the investigation |
|  | no medical history of dysplastic nevi or melanoma, of abnormal response to sunlight |

**Figure AF 1**:

**Figure title:** Evaluation of absorption time.


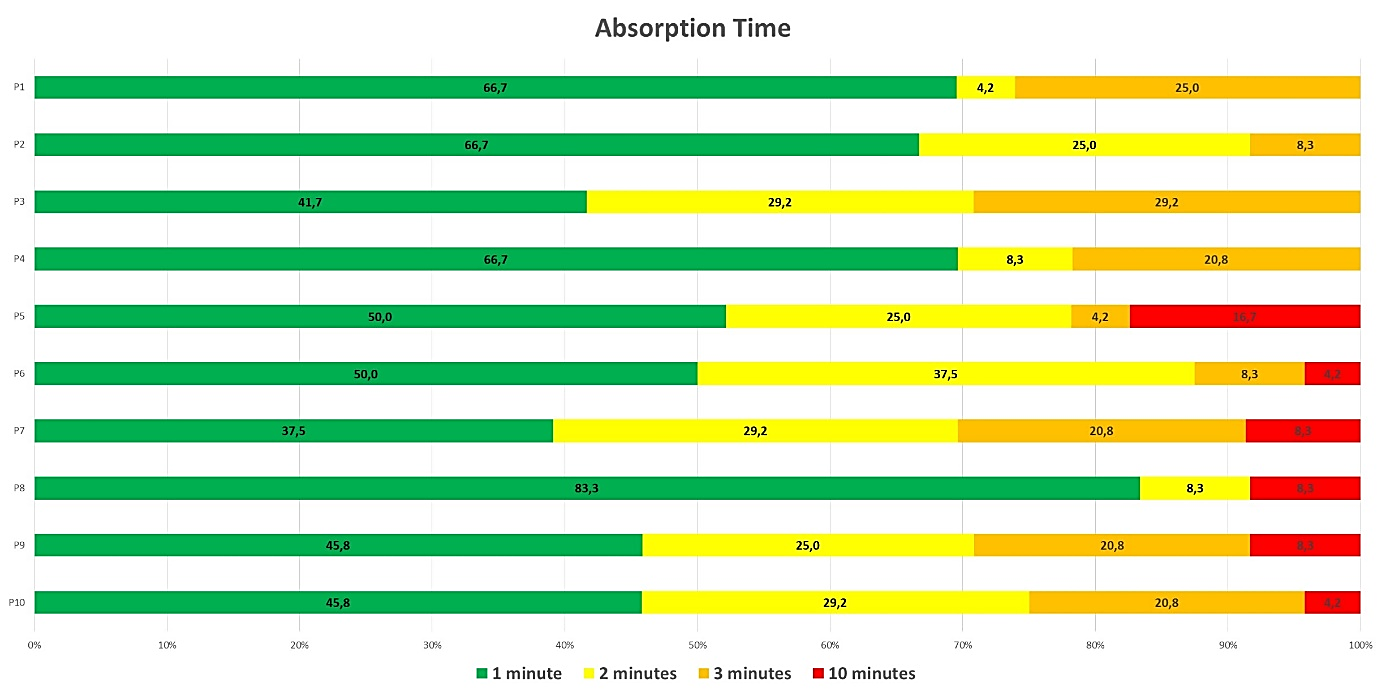


**Figure legend**: Absorption time was assessed 1, 2, and 3 minutes after test product application and, if applicable, 10 minutes after application, if not yet absorbed after 3 minutes. Data show percentages of “yes” ratings at assessment time. Single subjects stated an absorption time of 10 minutes regarding products P5 – P 10 while none of the subjects stated an absorption time longer than 3 minutes for codes P1, P2, P3 and P4. The shortest time to be absorbed was found for code P8.

**Figure AF 2**:

**Figure title:** Evaluation of subjective evaluation of skin feeling on wood.


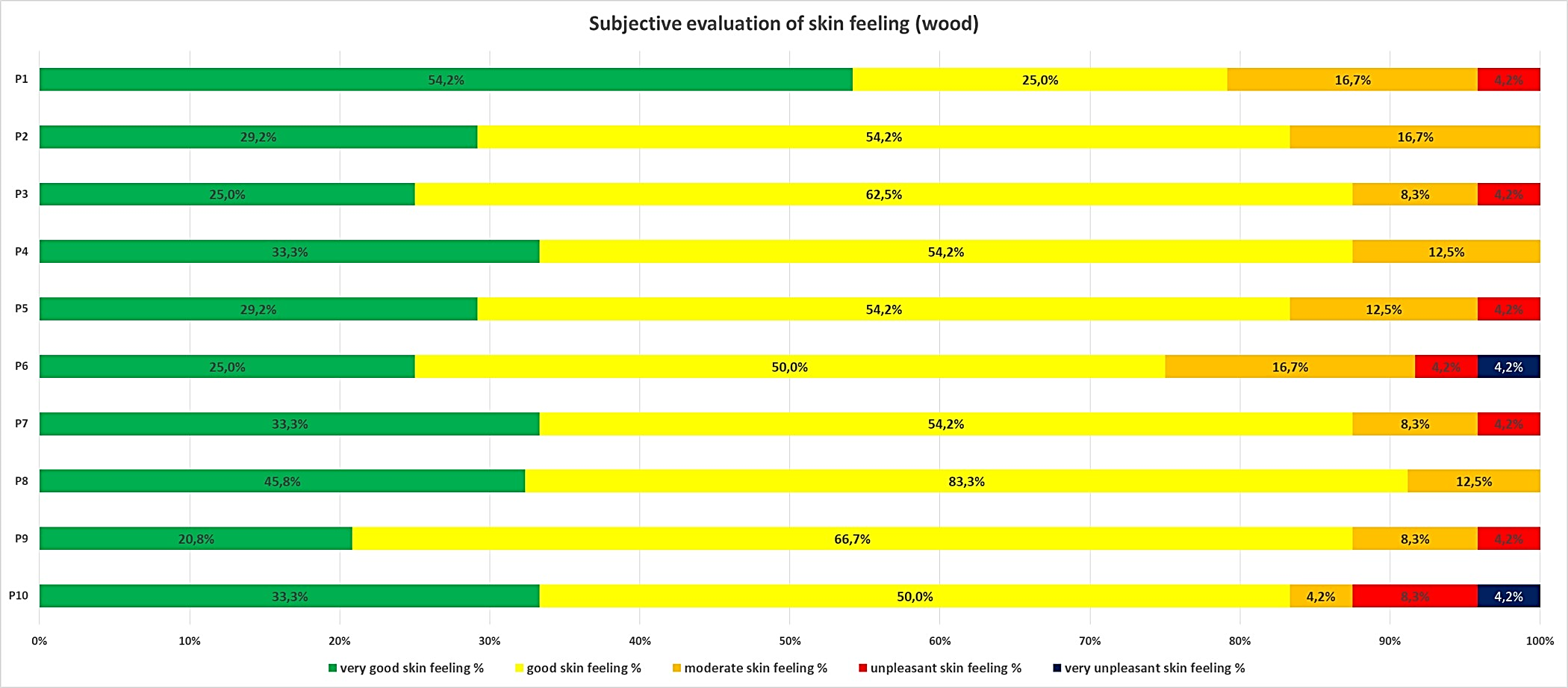


**Figure legend**: Subjective evaluation of skin feeling on a wooden bar after an absorption time of 3 minutes. Participants rated subjective skin feeling on a 5-point Likert scale. *Favorable*: Includes the answers for "very good skin feeling", "good skin feeling" and "moderate skin feeling". *Unfavorable*: Includes the answers for "unpleasant skin feeling" and "very unpleasant skin feeling".

**Figure AF 3**:

**Figure title:** Evaluation of subjective evaluation of skin feeling on metal.


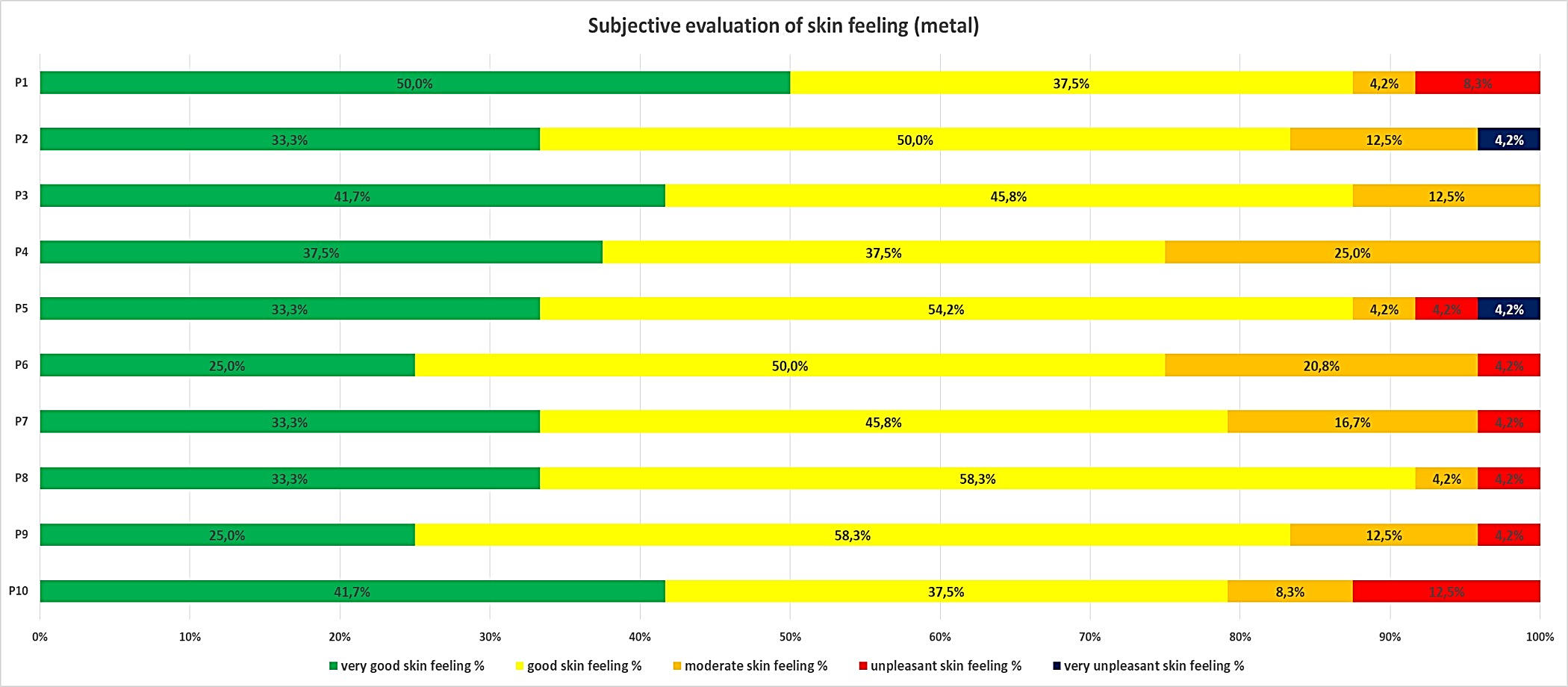


**Figure legend**: Subjective evaluation of skin feeling on a metal bar after an absorption time of 3 minutes. Participants rated subjective skin feeling on a 5-point Likert scale. *Favorable*: Includes the answers for "very good skin feeling", "good skin feeling" and "moderate skin feeling". *Unfavorable*: Includes the answers for "unpleasant skin feeling" and "very unpleasant skin feeling".

**Figure AF 4**:

**Figure title:** Evaluation of non-slip grip on wood.


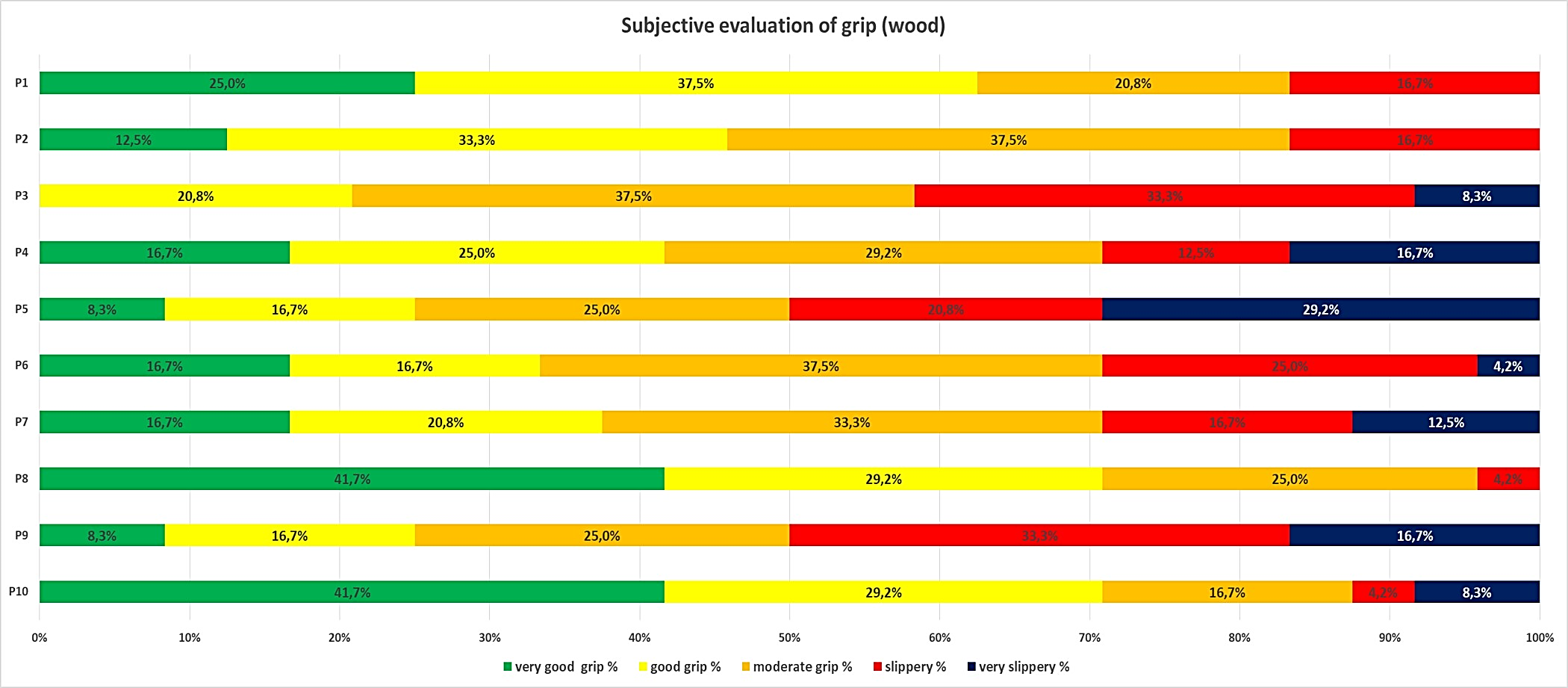


**Figure legend**: Subjective evaluation of non-slip grip on a wooden bar after an absorption time of 3 minutes. Participants rated subjective grip on a 5-point Likert scale. The best assessment for grip 3 minutes after product application was achieved by product P8 (about 96% favorable answers). Lowest frequencies of favorable answers were obtained for products P5, P9 and P3 (between 50% and 58% favorable answers). *Favorable*: Includes the answers for "very good grip", "good grip" and "moderate grip". *Unfavorable*: Includes the answers for "slippery" and "very slippery".

**Figure AF 5**:

**Figure title:** Evaluation of non-slip grip on metal.


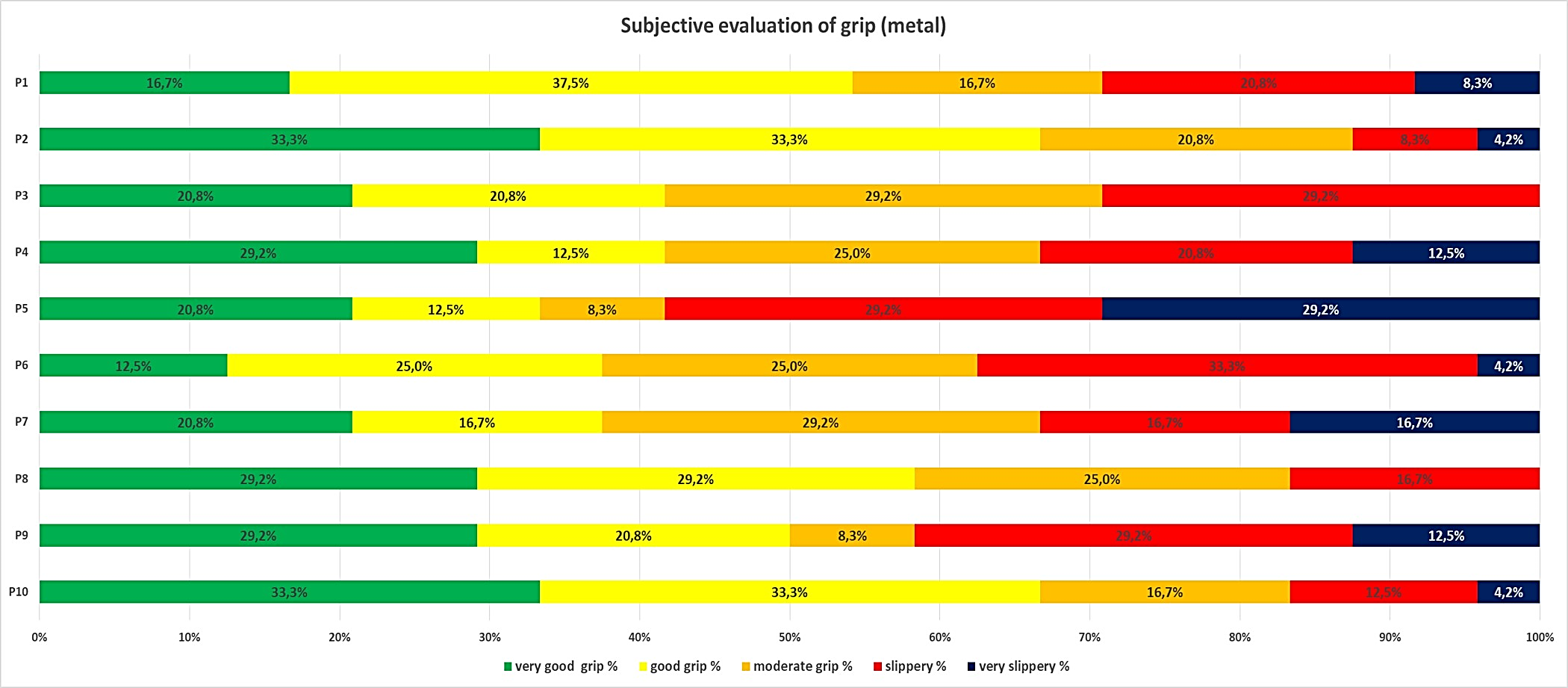


**Figure legend**: Subjective evaluation of non-slip grip on a metal bar after an absorption time of 3 minutes. Participants rated subjective grip on a 5-point Likert scale. The best assessment for grip 3 minutes after product application was achieved by product P2 (about 88% favorable answers). Lowest frequencies of favorable answers were obtained for products P5 and P9 (between 42% and 58% favorable answers). *Favorable*: Includes the answers for "very good grip", "good grip" and "moderate grip". *Unfavorable*: Includes the answers for "slippery" and "very slippery".
